# Hospital-wide SARS-CoV-2 antibody screening of staff in a university psychiatric centre in Belgium

**DOI:** 10.1192/bjo.2020.172

**Published:** 2021-01-20

**Authors:** Kawtar El Abdellati, Violette Coppens, Jobbe Goossens, Heidi Theeten, Pierre Van Damme, Ann Berens, Manuel Morrens, Livia De Picker

**Affiliations:** Collaborative Antwerp Psychiatric Research Institute, Faculty of Medicine and Health Sciences, University of Antwerp, Belgium; and Department of Psychiatry, Belgium; Collaborative Antwerp Psychiatric Research Institute, Faculty of Medicine and Health Sciences, University of Antwerp, Belgium; and Department of Psychiatry, University Psychiatric Hospital Campus Duffel, Belgium; Collaborative Antwerp Psychiatric Research Institute, Faculty of Medicine and Health Sciences, University of Antwerp, Belgium; and Department of Psychiatry, University Psychiatric Hospital Campus Duffel, Belgium; Centre for the Evaluation of Vaccination, Vaccine and Infectious Disease Institute, University of Antwerp, Belgium; Centre for the Evaluation of Vaccination, Vaccine and Infectious Disease Institute, University of Antwerp, Belgium; Department of Psychiatry, University Psychiatric Hospital Campus Duffel, Belgium; Collaborative Antwerp Psychiatric Research Institute, Faculty of Medicine and Health Sciences, University of Antwerp, Belgium; and Department of Psychiatry, University Psychiatric Hospital Campus Duffel, Belgium; Collaborative Antwerp Psychiatric Research Institute, Faculty of Medicine and Health Sciences, University of Antwerp, Belgium; and Department of Psychiatry, University Psychiatric Hospital Campus Duffel, Belgium

**Keywords:** Psychiatric nursing, COVID-19, serology testing, mental health services, infection prevention

## Abstract

In this first serosurvey among psychiatric healthcare providers, only 3.2% of a sample of 431 staff members of a Belgian University Psychiatric Centre, screened 3–17 June 2020, had SARS-CoV-2 immunoglobulin G antibodies, which is considerably lower compared with both the general population and other healthcare workers in Belgium. The low seroprevalence was unexpected, given the limited availability of personal protective equipment and the high amount of COVID-19 symptoms reported by staff members. Importantly, exposure at home predicted the presence of antibodies, but exposure at work did not. Measures to prevent transmission from staff to patients are warranted in psychiatric facilities.

Belgium has been severely affected by the COVID-19 pandemic.^1^ Compared with general hospitals, psychiatric hospitals and care facilities were disadvantaged in the Belgian governmental pandemic response. Governance of Belgian mental healthcare facilities is divided between federal and regional governments, which hampered early action and coordinated initiatives. Moreover, psychiatric facilities were poorly supplied with personal protective equipment (PPE) and nasopharyngeal swab testing kits compared with general hospitals.

The University Psychiatric Centre Duffel (UPC Duffel) is the largest tertiary psychiatric hospital in Belgium's Antwerp region, with a catchment area of 1.9 million residents. It has a capacity of 541 hospital beds and long-stay psychiatric care for an additional 90 patients. Throughout the pandemic, the hospital sustained its provision of residential psychiatric care and admitted new patients based on its regular referral criteria. Infection prevention measures were installed, including reverse transcription polymerase chain reaction (RT-PCR) nasopharyngeal swab testing of all newly admitted and symptomatic patients, restrictions in patients’ contact with the outside, and the instalment of a new unit to isolate psychiatric patients with COVID-19. Because of shortages in masks, gloves and robes, PPE was not routinely worn by either staff or patients, except for personnel taking care of patients isolated with suspected or confirmed COVID-19 diagnosis. The hospital's COVID-19 isolation unit was opened after the first PCR-positive case on 6 April 2020, and closed on 11 May 2020 (Fig. 1). A total of 18 psychiatric patients with COVID-19 were treated at the UPC Duffel. The aim of this study was to investigate the prevalence of antibodies against SARS-CoV-2 among hospital staff.

**Fig. 1:**
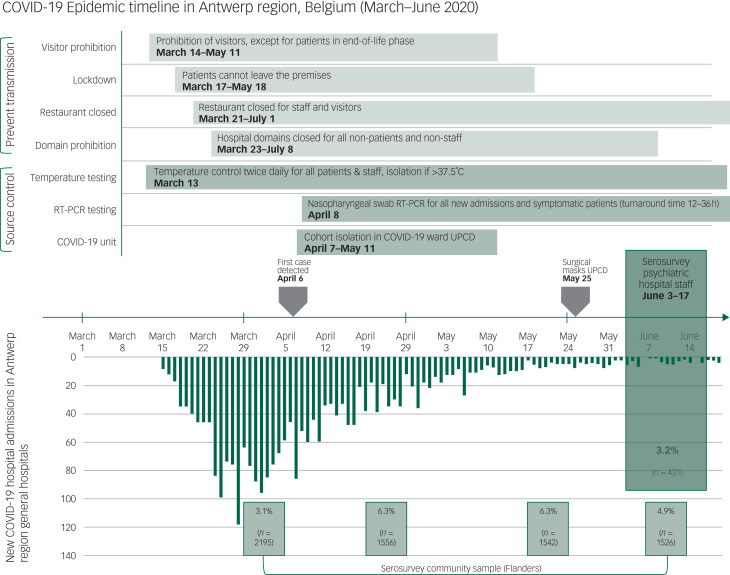
Epidemic timeline featuring the timing of source control and transmission prevention measures in the psychiatric hospital University Psychiatric Centre Duffel, as well as the incidence of new COVID-19 hospital admissions in the Antwerp region (source: Sciensano https://epistat.wiv-isp.be/covid/covid-19.html) and community seroprevalence results across four sampling windows: 30 March to 5 April, 20–26 April, 18–24 May and 8–14 June (details available from the authors on request). Surgical masks were not supplied to University Psychiatric Centre Duffel before 25 May 2020, and the first confirmed COVID-19 case was identified on 6 April 2020. RT-PCR, Reverse transcription polymerase chain reaction; UPCD, University Psychiatric Centre Duffel.

## Method

From 3 to 17 June 2020, both clinical and non-clinical staff at the UPC Duffel and associated long-stay facilities (PVT Schorshaegen) were invited for serological testing. Staff with active symptoms who were currently on sick leave could not be included. Serum samples were analysed with enzyme-linked immunosorbent assay (ELISA; Euroimmun AG, Luebeck, Germany) for SARS-CoV-2 immunoglobulin G (IgG) at the Antwerp Medical Laboratory, with a cut-off value ≥0.80. The manufacturer reported a sensitivity for IgG of 80% at >10 days after symptom onset, and a specificity of 98.5%. Demographic characteristics and job title were recorded, and staff were asked to complete a survey on exposure risks and symptoms from 15 February 2020 onward. The seroprevalence 95% confidence interval was calculated by the asymptotic method, and compared with the community seroprevalence in a 2 × 2 *χ*^2^-test (adjusted to an ELISA cut-off value ≥1.10). Odds ratios were calculated with bivariable logistic regression to assess demographic and job characteristics associated with seroprevalence, and with multivariable logistic regression to assess symptoms independently associated with seroprevalence, with all 14 symptoms included as covariates. One patient with missing IgG data was excluded from analysis. Statistical significance was set at a two-sided *P*-value <0.05. Analyses were performed in JMP® Pro Version 14.3.0 for Windows (64-bit) (SAS Institute Inc., Cary, NC, USA; see https://www.jmp.com).

The authors assert that all procedures contributing to this work comply with the ethical standards of the relevant national and institutional committees on human experimentation and with the Helsinki Declaration of 1975, as revised in 2008. All procedures involving human subjects were approved by the ethical committee of the University of Antwerp/University Hospital Antwerp (EC UZA/UA 20/16/210; Belgian number B3002020000067). Written informed consent was obtained for all participants.

## Results

A total of 397 (47.4%) out of 837 invited UPC Duffel staff members participated in the study (55.4% of total clinical staff, 36.7% of non-clinical staff). An additional 34 staff members from two associated long-stay residential facilities were also included. The sample comprised 18 physicians (18 of 18 physicians at UPC Duffel), 181 nurses (170 of 302 nurses at UPC Duffel), 26 psychologists (25 of 34 psychologists at UPC Duffel), 56 paramedical staff members (including social workers, non-verbal therapists, and physiotherapists; 41 of 75 staff at UPC Duffel), 135 nonclinical staff (housekeeping, technical and administrative; 128 of 353 staff at UPC Duffel) and 15 other staff members.

Overall, 14 staff members (3.2%; 95% CI, 1.9%–5.4%) were positive for IgG SARS-CoV-2 antibodies. Age and gender were not statistically significantly different among staff with or without antibodies (mean age, 46.8 ± 13.1 *v*. 43.7 ± 11.7 years; 71.4% *v*. 82.7% female). Being involved in clinical care, being involved in care for patients with COVID-19, working in emergency/acute psychiatry units, working in old-age psychiatry units and exposure to COVID-19 ( positive or suspected-positive co-workers) were not statistically significantly associated with seropositivity. Only a household contact with suspected or confirmed COVID-19 (*n* = 21) was significantly associated with antibody positivity, with an odds ratio of 7.3 (95% CI, 1.7–32.2; *P* = 0.008). Two out of the 14 staff with antibodies had previously tested positive on PCR.

Over a fifth (22.0%) of participants said they thought they had been infected with COVID-19 (excluding those with prior positive PCR), without significant difference between clinical and nonclinical staff. Participants with self-indicated suspected infection were significantly more likely to have COVID-19 antibodies (9.6% *v*. 0.9%; odds ratio, 11.6; 95% CI, 3.1–43.7; *P* < 0.001). Around half (46.5%) of participants mentioned at least one prior symptom of COVID-19 (pharyngitis/throat pain, 23.4%; cough, 21.1%; headache, 20.9%; rhinorrhoea, 19.7%; lethargy, 16.5%; myalgia/arthralgia/thoracic pain, 10.0%; dyspnoea, 8.6%; chills/feverish malaise, 8.4%; diarrhoea, 7.2%; fever, 6.3%; nausea/vomiting, 2.6%; anosmia/ageusia, 2.6%; skin rash, 1.9%; irritability/confusion, 1.4%). Of those with antibodies, 5 of 14 (35.7%) reported no prior symptoms. Only prior anosmia/ageusia (odds ratio, 17.3; 95% CI, 2.3–127.2; *P* = 0.005) and fever (odds ratio, 14.8; 95% CI 2.3–93.7; *P* = 0.004) were significantly associated with the presence of antibodies.

## Discussion

Psychiatric staff seroprevalence was surprisingly not increased compared with the general population in the Flanders region (adjusted seroprevalence, 3.0% *v*. 4.9%; *χ*² = 2.79; *P* = 0.095; Fig. 1), and even decreased compared with the Belgian population seroprevalence (3.0% *v*. 5.5%, *χ*² = 4.73; *P* = 0.030).^2^ This finding was unexpected, as the psychiatric hospital continued its activities and was unable to implement routine use of PPE throughout most of the pandemic. Previous studies have indicated a higher seroprevalence among Belgian general hospital staff. Steensels et al reported a 6.4% seroprevalence among 3056 staff of a third-line general hospital sampled 22–30 April 2020.^3^ Another study among 834 healthcare providers from 17 Belgian hospitals screened 19–24 May 2020 reported a 8.8% seroprevalence.^4^ The relatively low seroprevalence in our study might be explained by early implementation of source control and transmission prevention (Fig. 1) and/or as a consequence of waning antibodies (measurement in June), after a longer interval since exposure in March or April.^5^ However, all previously symptomatic staff members with antibodies reported having had COVID-19-related symptoms in March 2020, in the beginning of the epidemic. As we did not assess symptom severity, it is unclear to which extent early or mild cases, who may not generate anti-SARS-Cov-2 antibodies, were missed in this serosurvey. The same applies to, on average, 14 staff members who were on sick leave during our sampling window. Other limitations of this study include the single-centre design and testing of only 47.4% of staff, although all of the different staff functions were well represented in the sample.

This study represents the first hospital-wide screening for SARS-CoV-2 antibodies among psychiatric staff. Hospital-wide antibody screening can help evaluate transmission dynamics and infection control policies. Importantly, neither being involved in clinical care nor having been exposed to patients or co-workers with COVID-19 increased the odds of being seropositive, whereas having a suspected COVID-19-positive household contact did. These findings are in line with those previously reported among staff of a general hospital in Belgium,^3^ and indicate that, similar to what has been described in elderly care homes, staff being exposed to COVID-19 outside of the workplace could represent a source of infection in psychiatric facilities. Our data corroborate the findings from other authors that although patients with mental disorders are at increased risk of COVID-19 infection,^6,7^ with appropriate precautions, in-patient psychiatric services do not represent COVID-19 outbreak hotspots, and even patients with COVID-19 who need in-patient psychiatric treatment can be managed safely.^8,9^

## Data Availability

The data that support the findings of this study are available from the corresponding author, L.D.P., upon reasonable request.

## Supplementary material

For supplementary material accompanying this paper visit https://doi.org/10.1192/bjo.2020.172. click here to view supplementary material
